# Transcriptomics Reveals Cold Tolerance Maize Lines Involved in the Phenylpropanoid and Flavonoid Pathways

**DOI:** 10.3390/plants15010161

**Published:** 2026-01-05

**Authors:** Shuna Zhou, Xinling Yu, Jian Tan, Haixiao Sun, Wei Yang, Liangyu Jiang, Zhenyuan Zang, Jiabin Ci, Xuejiao Ren

**Affiliations:** College of Agriculture, Jilin Agricultural University, Changchun 130118, China; zsn16655805901@163.com (S.Z.); yxl18654060901@163.com (X.Y.); 18097479424@163.com (J.T.); 17767764577@163.com (H.S.); davidyoung588@126.com (W.Y.); liangyu0113@163.com (L.J.); zhenyuanzang1989@163.com (Z.Z.)

**Keywords:** maize germination, low-temperature stress, phenylpropanoid biosynthesis, flavonoid biosynthesis, transcriptomics, ROS scavenging, lignin biosynthesis

## Abstract

Low temperature during early spring severely impairs maize germination, leading to significant yield losses. To elucidate the mechanisms underlying cold tolerance at the germination stage, we compared two cold-tolerant maize inbred lines (AM and CM) with a cold-sensitive line (BM) under control (25 °C) and chilling (6 °C) conditions. Phenotypic observations showed that AM and CM maintained high germination rates and exhibited enhanced coleoptile elongation under cold stress, whereas BM displayed substantial growth inhibition. Cold-tolerant lines accumulated less malondialdehyde and showed markedly higher SOD and POD activities, indicating a stronger antioxidant defense. Transcriptome profiling revealed that cold tolerance is associated with a more robust transcriptional response in AM and CM, characterized by significant activation of the phenylpropanoid and flavonoid biosynthesis pathways. Among the differentially expressed genes, the class III peroxidase gene *ZmPER5* was strongly upregulated in AM and CM but only weakly induced in BM, suggesting its central role in reinforcing the cell wall structure and enhancing ROS-scavenging capacity under chilling conditions. Other lignin- and flavonoid-related genes, including *ZmHCT4* and *ZmCYP75*, also exhibited genotype-specific induction patterns consistent with cold tolerance. qRT-PCR validation confirmed the RNA-seq expression trends. These results demonstrate that maize cold tolerance during germination relies on the coordinated enhancement of antioxidant enzyme activity, activation of phenylpropanoid-derived lignin biosynthesis, and accumulation of protective flavonoids. The identified candidate genes, especially *ZmPER5*, provide valuable targets for improving cold tolerance in maize breeding.

## 1. Introduction

Maize (*Zea mays* L.) is the world’s third most widely grown cereal crop and a major source of food, feed, and bioenergy, contributing substantially to global food security and agricultural sustainability [1,2]. However, in temperate and cool regions, early spring low-temperature stress is a major constraint to maize establishment. Chilling conditions during germination significantly delay seedling emergence, impair metabolic activity, and ultimately reduce yield, causing estimated annual global losses of 12–15% [3,4].

Low temperature disrupts key physiological processes, including energy metabolism, antioxidant activity, and membrane stability. It reduces membrane fluidity, triggers excessive reactive oxygen species (ROS) accumulation, and induces lipid peroxidation, collectively leading to inhibited radicle and coleoptile elongation and high seedling mortality [5,6]. Coleoptile growth is particularly sensitive to chilling stress, and its elongation ability has been widely recognized as a rapid and reliable phenotypic indicator of low-temperature tolerance in maize and other cereals [7,8,9].

Cold tolerance during germination is controlled by complex genetic and metabolic networks. Several studies have identified cold-responsive loci through genome-wide association studies (GWASs) in rice and maize [10,11]. In maize, cold-induced alterations in coleoptile and mesocotyl growth have also been linked to light quality, hormonal balance, and transcriptional regulation [12]. At the molecular level, cold stress activates signaling pathways involving calcium-dependent protein kinases (CDPKs) and mitogen-activated protein kinases (MAPKs), which modulate downstream transcription factors and stress-responsive genes [13,14,15]. Furthermore, antioxidant enzymes such as SOD, POD, and APX play essential roles in ROS detoxification, and their activities are closely associated with chilling tolerance in maize and other crops [16,17,18].

Recent studies highlight the importance of secondary metabolism, particularly the phenylpropanoid pathway, in plant cold responses. This pathway produces lignin, flavonoids, and related metabolites that participate in cell wall reinforcement and non-enzymatic antioxidant defense [19]. In maize, cold stress has been shown to modulate phenylpropanoid and flavonoid accumulation in radicles and seedlings, suggesting a key role in cold tolerance [20,21]. However, most studies to date have focused on individual pathways, specific developmental stages, or isolated genotypes, and the integrated molecular mechanisms linking phenylpropanoid activation, antioxidant capacity, and coleoptile elongation during germination remain insufficiently understood.

To address these gaps, we investigated two cold-tolerant maize inbred lines (AM, CM) and one cold-sensitive line (BM) subjected to control and chilling conditions. By integrating phenotypic, physiological, and transcriptomic analyses, we aimed to (i) characterize genotype-specific responses to cold stress during germination, (ii) identify key pathways and genes associated with cold tolerance, and (iii) determine how phenylpropanoid and flavonoid biosynthesis contribute to the enhanced coleoptile growth observed in tolerant lines. Our findings provide new mechanistic insights and potential molecular targets for improving cold resilience in maize breeding.

## 2. Results

### 2.1. Low Temperature Differentially Affects Germination and Coleoptile Growth in Three Maize Inbred Lines

**Figure 1:**
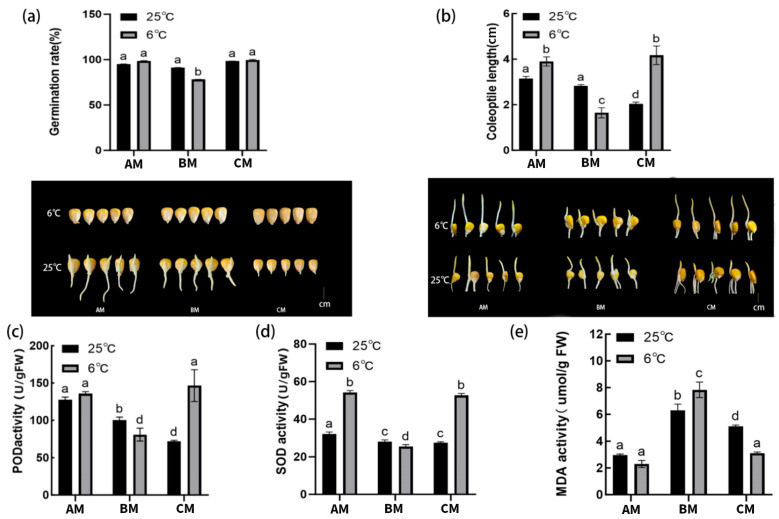
Phenotypic performance and oxidative stress-related physiological responses of three maize inbred lines (AM, BM, and CM) under control (25 °C) and low-temperature (6 °C) conditions during germination. (**a**) Germination rate (%) after 3 days of incubation at 25 °C or 6 °C, showing genotype-dependent sensitivity to low temperature. (**b**) Coleoptile length of germinated seedlings under control and cold stress; representative images of seedlings are shown below the bar chart to illustrate visual differences in early growth vigor. (**c**) Peroxidase (POD) activity. (**d**) Superoxide dismutase (SOD) activity. (**e**) Malondialdehyde (MDA) content. Values are presented as mean ± SD (n = 3 biological replicates). Different lowercase letters indicate statistically significant differences among genotypes and temperature treatments (*p* < 0.05).

Cold stress induced clear and genotype-dependent variations in early seedling performance (Figure 1a,b). Under control conditions (25 °C), the three inbred lines exhibited high germination rates (91–100%). However, exposure to 6 °C significantly reduced germination in the cold-sensitive line BM (*p* < 0.05), whereas AM and CM maintained high germination (>95%) without significant decline (Figure 1a). Coleoptile elongation displayed even stronger genotype differentiation (Figure 1b). BM showed a 50% reduction in coleoptile length at 6 °C (3.60 → 1.80 cm), confirming its chilling sensitivity. In contrast, AM and CM exhibited increased coleoptile elongation under cold stress, with CM showing the largest response (2.70 → 4.05 cm), indicating enhanced early growth vigor. Representative seedling images visually confirm these phenotypic differences.

### 2.2. Low Temperature Induces Genotype-Dependent Changes in Oxidative Stress-Related Physiological Parameters

Low temperature substantially affected membrane integrity and redox homeostasis (Figure 1c–e). MDA content increased sharply in BM (4.92 → 7.85 μmol g^−1^ FW), indicating enhanced lipid peroxidation, whereas AM and CM maintained significantly lower MDA levels (*p* < 0.05), reflecting better membrane stability (Figure 1e). Antioxidant enzyme activities showed contrasting trends among the genotypes. SOD and POD activities increased significantly in AM and CM under cold stress, with CM exhibiting the highest enzyme induction (Figure 1c,d). In contrast, BM showed no significant increase in SOD activity and even reduced POD activity. These results indicate that tolerant lines possess stronger ROS-scavenging capacity, consistent with their phenotypic resilience.

### 2.3. Transcriptome Profiling Reveals Strong Genotype- and Temperature-Dependent Transcriptional Reprogramming

**Figure 2:**
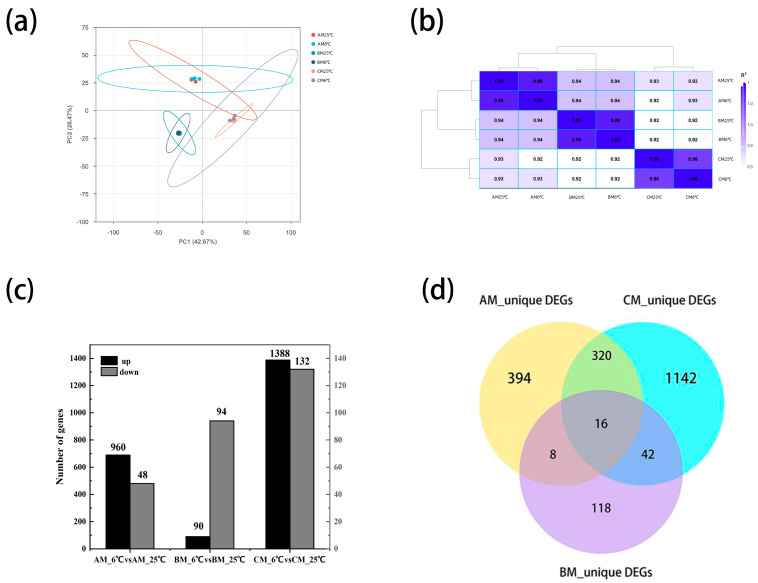
Global transcriptomic responses of maize coleoptiles to low-temperature stress in AM, BM, and CM lines. (**a**) Principal component analysis (PCA) of all transcriptome samples based on normalized gene expression levels. Each point represents one biological replicate, and the separation among samples reflects differences in overall transcriptional profiles driven by genotype and temperature treatment. (**b**) Pearson correlation heatmap showing the similarity of transcript expression patterns among all samples, demonstrating high reproducibility between biological replicates. (**c**) Number of upregulated and downregulated differentially expressed genes (DEGs) identified in each inbred line under low-temperature treatment (6 °C) compared with the corresponding control (25 °C). (**d**) Venn diagram showing the overlap and uniqueness of upregulated DEGs among AM, BM, and CM under cold stress. Shared DEGs represent common cold-responsive genes, whereas unique DEGs indicate genotype-specific transcriptional responses.

To investigate the molecular basis underlying cold responses, transcriptomic analyses were performed (Figure 2a–d). PCA clearly separated samples by temperature and genotype (Figure 2a), with strong intra-group correlation (Figure 2b). Cold stress triggered extensive transcriptional activation in the tolerant lines. CM exhibited the largest number of upregulated DEGs (1388), followed by AM (690), whereas BM showed significantly fewer DEGs (90), indicating a weaker transcriptional response (Figure 2c). Venn diagrams revealed 320 co-upregulated genes shared between AM and CM but largely absent in BM, suggesting a core cold-tolerance module (Figure 2d).

### 2.4. GO Enrichment Analysis Identifies Cold-Induced Activation of Oxidative Stress- and Ion Transport-Related Processes in Tolerant Lines

**Figure 3:**
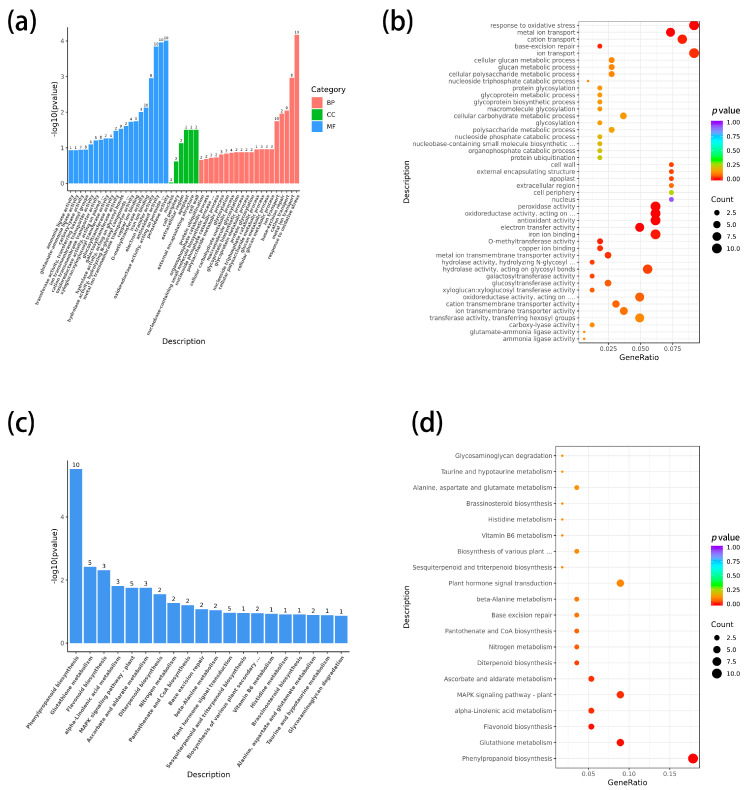
Functional characterization of cold-responsive genes shared by cold-tolerant maize lines. (**a**) Gene Ontology (GO) enrichment analysis of differentially expressed genes commonly induced by low-temperature stress in the cold-tolerant lines AM and CM, categorized into biological process, molecular function, and cellular component. (**b**) Bubble plot of significantly enriched GO terms, where bubble size indicates the number of genes and color represents the level of statistical significance. (**c**) Kyoto Encyclopedia of Genes and Genomes (KEGG) pathway enrichment analysis of shared DEGs, highlighting pathways significantly associated with cold stress response. (**d**) Bubble plot showing the top enriched KEGG pathways, illustrating the relative contribution and significance of each pathway in cold-tolerant genotypes.

GO enrichment of the 320 shared DEGs revealed significant enrichment in oxidative stress response, oxidoreductase activity, peroxidase activity, and cation/metal ion transport (Figure 3a,b). These enriched functions are consistent with the increased SOD/POD activities observed physiologically. Notably, peroxidase-related terms were strongly represented, consistent with the upregulation of class III peroxidases such as ZmPER5. Ion transport–related terms suggest strengthened metal homeostasis, potentially supporting antioxidant enzyme cofactor requirement (e.g., Cu/Zn-SOD).

### 2.5. KEGG Pathway Enrichment Shows Specific Activation of Phenylpropanoid and Flavonoid Biosynthesis in Cold-Tolerant Lines

**Figure 4:**
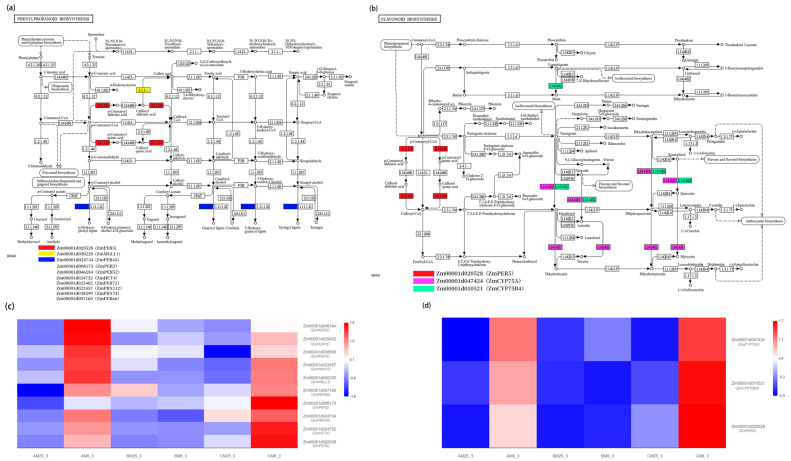
KEGG pathway enrichment and expression profiles of genes associated with phenylpropanoid and flavonoid biosynthesis under low-temperature stress. (**a**) KEGG phenylpropanoid biosynthesis pathway showing differentially expressed genes (DEGs) enriched in this pathway under low-temperature treatment. Colored boxes indicate DEGs identified by KEGG enrichment analysis, corresponding to the gene list shown in the legend. (**b**) KEGG flavonoid biosynthesis pathway showing DEGs enriched in the flavonoid biosynthesis pathway under low-temperature treatment. Colored boxes correspond to genes listed in the legend. (**c**) Heatmap of expression profiles of phenylpropanoid pathway–related genes across different sample groups defined by genotype (AM, BM, and CM) and temperature treatments (25 °C and 6 °C). Color intensity represents relative gene expression levels (blue, low expression; red, high expression). Sample labels indicate different genotype × temperature combinations. (**d**) Heatmap of expression profiles of flavonoid biosynthesis-related genes across the same sample groups. Color scale and sample definitions are the same as in panel (**c**).

We performed functional annotation and expression analysis of 320 differentially expressed genes (DEGs), with detailed annotations and FPKM expression values provided in Supplementary Tables S1–S3. Heatmap analysis revealed that these DEGs were generally highly expressed in the AC inbred line, suggesting that AC may enhance cold response through these genes (Figure 4c). KEGG enrichment analysis showed that phenylpropanoid biosynthesis and flavonoid biosynthesis were significantly enriched in the AM and CM inbred lines (Figure 3c,d). The specific pathways and genes that were significantly enriched are listed in Supplementary Table S4. In the pathway visualization (Figure 4a,b), we marked the positions of these DEGs to show their enrichment at specific nodes within the pathways, particularly in the key nodes related to lignin monomer synthesis and flavonoid metabolism. However, this enrichment does not imply that the entire pathway was globally activated, but rather that these specific nodes were enriched with differentially expressed genes. The heatmap also revealed that cold-tolerant AM and CM lines exhibited notable differential expression of key genes, including lignin-related genes (ZmHCT4), peroxidase genes (ZmPER5), and flavonoid biosynthesis genes. Notably, ZmPER5 was significantly enriched in both phenylpropanoid and flavonoid biosynthesis pathways, suggesting a central role in the cold response. In contrast, the BM inbred line showed minimal differences in these key genes, consistent with its lower cold tolerance. Overall, cold-tolerant inbred lines may enhance low-temperature adaptation through differential expression of key genes at specific nodes in lignin and flavonoid metabolic pathways, with ZmPER5 potentially serving as a key regulatory node in this network.

### 2.6. Validation of RNA-Seq Data by qRT-PCR

**Figure 5:**
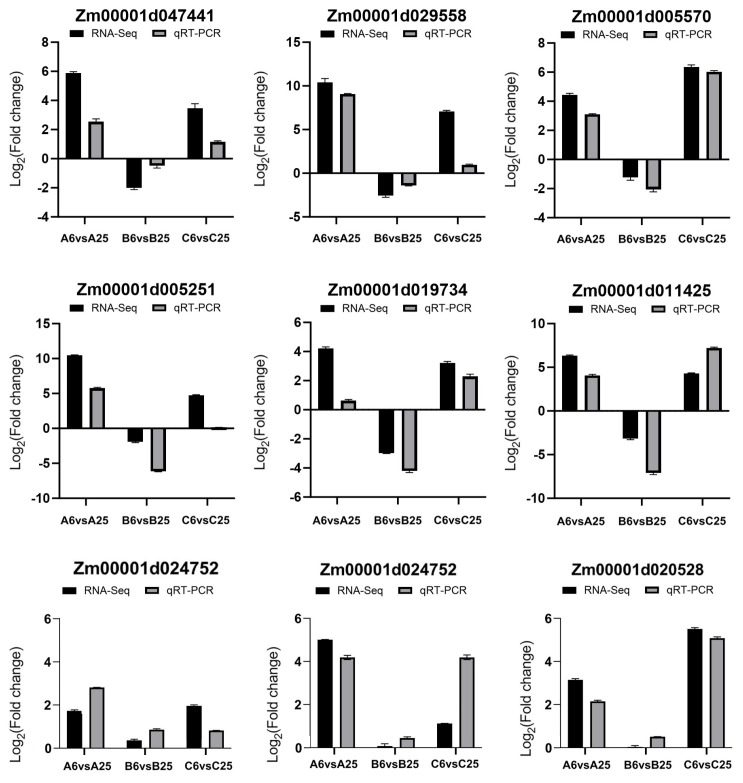
qRT-PCR validation of RNA-seq results. Comparison of log_2_ fold-change values obtained by RNA-seq and qRT-PCR for six representative genes across all genotype temperature comparisons.

The reliability of the transcriptome data was confirmed by quantitative real-time PCR (qRT-PCR). A subset of genes from the 320 differentially expressed genes (DEGs), including Zm00001d047441, Zm00001d029558, Zm00001d005570, Zm00001d005251, Zm00001d019734, Zm00001d011425, Zm00001d047424, Zm00001d024752, Zm00001d020528, were selected for validation across all sample groups. The expression trends (log2 fold change) detected by qRT-PCR were highly consistent with the RNA-Seq data (Figure 5), validating the identified DEGs.

## 3. Discussion

This study delineates the molecular mechanisms of low-temperature tolerance during maize germination by integrating phenotypic, physiological, and transcriptomic analyses of cold-tolerant (AM, CM) and cold-sensitive (BM) inbred lines.

### 3.1. Phenotypic and Physiological Distinctions Define Cold Tolerance

Under 6 °C stress, the cold-tolerant lines (AM and CM) exhibited markedly greater resilience than the sensitive BM line. The BM line displayed reduced germination (78.3%) and 31% coleoptile shortening. In contrast, the AM and CM lines maintained germination rates above 98%, with the CM line showing notable coleoptile elongation (a 50% increase). This phenotypic advantage was underpinned by a 20–50% decrease in malondialdehyde (MDA) content and significantly elevated activities of superoxide dismutase (SOD) and peroxidase (POD) in the tolerant lines, contrasting with the opposite trends in BM.

The capacity of our tolerant lines to maintain >95% germination at 6 °C aligns with the performance of cold-tolerant rice genotypes at 8 °C [22] and contrasts with the 10–25% germination loss reported for many maize varieties below 10 °C [23]. Furthermore, the paradoxical coleoptile elongation under cold stress mirrors the enhanced growth phenotype of Arabidopsis eskimo1 mutants at 4 °C [24]. This response represents a distinct adaptive strategy compared to the growth suppression typical in wheat under similar conditions.

### 3.2. Specific Activation of Phenylpropanoid and Flavonoid Biosynthesis Pathways Drives Adaptive Responses

Transcriptome analysis revealed the cold-specific activation of the phenylpropanoid (map00940) and flavonoid biosynthesis (map00941) pathways in the tolerant lines. This finding aligns with the reported upregulation of flavonoid-related metabolism under cold stress in maize [25]. However, our study extends this knowledge by directly linking the co-activation of these two pathways to the distinctive phenotypic trait of coleoptile elongation and by elucidating a key downstream structural adaptation. Key genes ZmPER5 and *ZmHCT4* were upregulated by 5-fold and 4-fold, respectively. *ZmPER5* exhibits dual functionality in antioxidant activity and phenylpropanoid metabolism regulation [26], consistent with research on cold-induced phenylpropanoid accumulation in winter cereals. This activation synergizes with the flavonoid-mediated antioxidant mechanism, analogous to the anthocyanin ROS scavenging pathway in Arabidopsis [27].

Beyond antioxidant roles, these pathways crucially contribute to structural adaptation. The concerted upregulation of phenylpropanoid biosynthesis genes, particularly those involved in lignin monomer synthesis (e.g., *ZmHCT4*) and polymerization (e.g., *ZmPER5*), indicates a reprogramming of cell wall metabolism. In maize, abiotic stresses such as salinity induce a coordinated “cell wall remodeling” response, where targeted deposition of lignin and other components reinforces structural integrity to mitigate stress damage while supporting continued growth [28]. Such spatially regulated lignification can fortify specific tissues (e.g., vascular bundles) without globally arresting organ elongation [29]. Therefore, we propose that in our cold-tolerant lines, the induced lignin biosynthesis is a component of adaptive cell wall fortification. This remodeling likely enhances the mechanical strength and integrity of the coleoptile under cold stress, protecting against cellular damage and sustaining turgor-driven cell expansion, thereby facilitating the observed organ elongation.

### 3.3. Coordinated Enhancement of the Integrated Antioxidant System

Redox homeostasis under cold was maintained by the coordinated enrichment of genes involved in oxidative stress response (GO:0006979), antioxidant activity (GO:0016209), and metal ion transport (GO:0030001). The upregulation of peroxidase genes showed a positive correlation with SOD/POD activities (r = 0.92). This finding corroborates studies linking robust antioxidant systems to cold tolerance in barley [30] and provides a transcriptional explanation for the physiological reduction in MDA. Concurrently, the induction of metal ion transporter genes (e.g., Zn^2+^/Cu^2+^ transporters) offers new evidence in maize for mechanisms ensuring SOD cofactor homeostasis under cold stress, a supporting mechanism also noted in rice [31].

### 3.4. Genotypic Variation in the Transcriptional Response and Implications

The scale of the transcriptional response to cold stress varied significantly between the tolerant genotypes. The CM line exhibited a far greater number of cold-responsive and uniquely expressed genes than AM, indicating that superior tolerance may be associated with a more expansive and potentially specialized gene regulatory network. This variation in the breadth and intensity of gene expression changes aligns with findings in other plants, where differences in cold signaling and regulation lead to distinct adaptive outcomes [32]. While our study confirms the involvement of conserved stress-response pathways, it also highlights that the degree and coordination of their transcriptional activation are key determinants of phenotypic resilience.

### 3.5. Synthesis and Mechanistic Model

**Figure 6:**
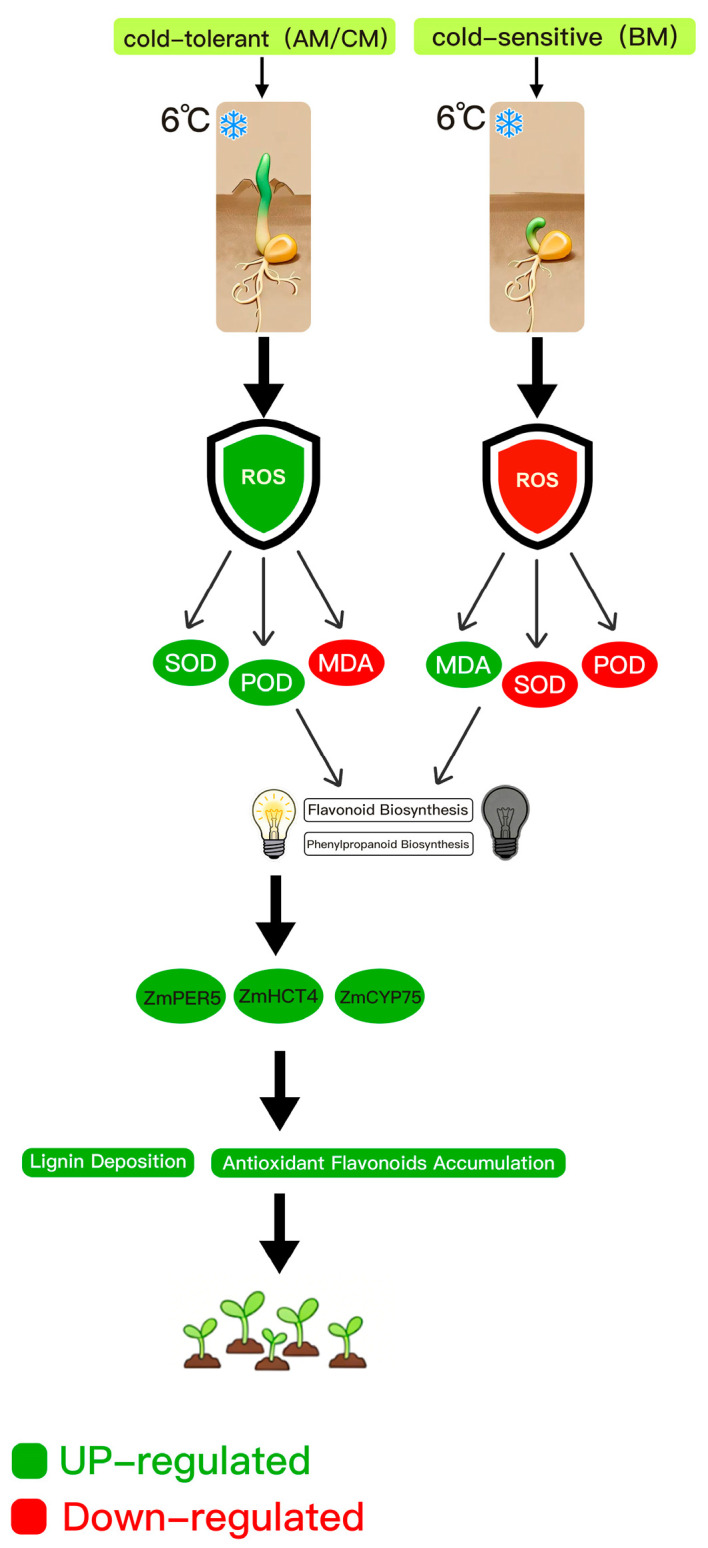
A proposed mechanistic model of cold tolerance in maize germination under 6 °C stress.

This study elucidates the molecular mechanisms of low-temperature tolerance during maize germination by integrating phenotypic, physiological, and transcriptomic analyses and proposes a synergistic model (Figure 6). The cold-tolerant inbred lines, especially CM, respond to low-temperature stress by activating a dual adaptive strategy: first, the phenylpropanoid pathway is specifically activated, driving the biosynthesis and accumulation of lignin and related metabolites; second, the flavonoid pathway enhances non-enzymatic antioxidant capacity, which works in concert with a strengthened enzymatic antioxidant system (SOD/POD) to effectively scavenge reactive oxygen species and maintain redox homeostasis. The superior cold tolerance of the CM line is attributed to its stronger and more coordinated activation of these key pathways, along with more extensive and systematic transcriptional reprogramming. These findings establish a mechanistic framework linking specific metabolic pathways to the crucial early-vigor trait of coleoptile elongation, providing valuable targets for molecular breeding of cold-resilient maize.

## 4. Materials and Methods

### 4.1. Plant Materials

Three maize (*Zea mays* L.) inbred lines with previously characterized contrasting cold tolerance were used: J1898 (cold-tolerant, designated AM in this study), J1218 (cold-tolerant, designated CM), and PH4CV (cold-sensitive, designated BM). Seeds were provided by the Maize Breeding Innovation Team, College of Agriculture, Jilin Agricultural University, Changchun, China.

### 4.2. Seed Germination Assay

Seed germination was evaluated using the rolled paper method [32] in a completely randomized block design. For each inbred line and temperature treatment (25 °C or 6 °C), three biological replicates were performed, each consisting of 100 surface-sterilized seeds on 40 × 50 cm germination paper. Seeds were placed with ventral grooves downward and embryos upward, and the papers were rolled and incubated vertically at the corresponding temperature. Germination was recorded daily, and a seed was considered germinated when the radicle protruded ≥2 mm beyond the seed coat, totaling 300 seeds per line per treatment [33].

### 4.3. Germination Rate and Coleoptile Length Measurement

During the germination period, seeds were observed daily. On day 3, germination status was recorded using the criterion of radicle protrusion ≥2 mm beyond the seed coat. The number of germinated seeds was counted, and coleoptile length was measured using a ruler [34].

### 4.4. Physiological Index Determination

#### 4.4.1. SOD Activity Assay

SOD activity was determined using the nitroblue tetrazolium (NBT) photochemical reduction method [35]. The reaction mixture contained:1.5 mL of 0.05 M phosphate buffer (pH 7.8); 0.3 mL of 180 μM methionine; 0.3 mL of 750 μM NBT; 0.3 mL of 100 μM EDTA-Na_2_; 0.3 mL distilled water; 0.05 mL enzyme extract. After thorough mixing, reactions were illuminated at 4000 lux for 10 min. Absorbance was immediately measured at 560 nm using a spectrophotometer. Blank controls contained phosphate buffer instead of enzyme extract. Three biological replicates were performed.

#### 4.4.2. POD Activity Assay

POD activity was measured using the guaiacol method [36]. The reaction was initiated by adding 1 mL of crude POD extract to 3 mL of reaction mixture (containing 25 mM guaiacol and 10 mM H_2_O_2_ in 50 mM phosphate buffer, pH 6.0). Absorbance changes at 470 nm were recorded at 1-min intervals for 3 min using a spectrophotometer. Blank controls lacked enzyme extract. Three biological replicates were performed.

#### 4.4.3. Malondialdehyde (MDA) Content Determination

MDA content was quantified via the thiobarbituric acid (TBA) reaction [37]. MDA reacts with TBA to form a red adduct with maximum absorbance at 532 nm. Absorbance at 532 nm was measured spectrophotometrically and MDA concentration calculated using an extinction coefficient of 155 mM^−1^cm^−1^. Three technical replicates were performed per sample.

### 4.5. Transcriptome Sequencing

#### 4.5.1. RNA Sequencing and Data Processing

Coleoptile tissues from three inbred lines under 6 °C and 25 °C treatments were harvested from rolled paper germination assays for RNA extraction [38]. Samples were collected from 3-day-old coleoptiles, immediately flash-frozen in liquid nitrogen, and stored at −80 °C. Total RNA was isolated from coleoptiles using TRIzol reagent (Invitrogen, Carlsbad, CA, USA, cat. no. GT0240) with three biological replicates per treatment group. RNA integrity was verified using an Agilent 2100 Bioanalyzer (Agilent Technologies, Santa Clara, CA, USA), and only samples with an RNA Integrity Number (RIN) ≥ 8.0 were selected for subsequent experiments. Strand-specific cDNA libraries were prepared using the NEBNext Ultra II RNA Library Prep Kit (New England Biolabs, Ipswich, MA, USA, cat. no. E7775L) [39]. Libraries were quantified with Qubit 3.0 and sequenced on the Illumina NovaSeq 6000 platform (150-bp paired-end reads; ≥20 million reads per sample).

Raw data were processed using Trimmomatic v0.39 to remove adapter sequences and low-quality bases (Phred score < 20). High-quality filtered reads were aligned to the maize B73 reference genome (version 4) using HISAT2 v2.2.1 with default parameters [40].

#### 4.5.2. Differential Gene Expression Analysis

Differentially expressed genes (DEGs) were identified using DESeq2 (v1.20.0) with thresholds of |log_2_(fold change)| > 1 and a false discovery rate (FDR) < 0.05 [41]. Comparisons were performed for each inbred line (AM, BM, CM) between cold-treated (6 °C) and control (25 °C) groups. Additional comparisons examined differences between cold-tolerant (AM/CM) and cold-sensitive (BM) germplasms under cold stress. Gene Ontology (GO) enrichment analysis was conducted using clusterProfiler with significance threshold of *p* < 0.05. Gene Set Enrichment Analysis (GSEA) was performed using the local GSEA tool (http://www.broadinstitute.org/gsea/index.jsp) against maize-specific GO and KEGG datasets.

#### 4.5.3. Quantitative Real-Time PCR (qRT-PCR) Validation

Key differentially expressed genes were validated using SYBR Green-based quantitative PCR [42]. Total RNA was extracted from plant tissues using TRIzol reagent (Invitrogen, cat. no. GT0240) [43]. First-strand cDNA synthesis was performed with 1 μg RNA using the ReverTra Ace qPCR RT Kit (TOYOBO, Shanghai, China; Code No. FSQ-101). qPCR reactions were conducted using 2× RealStar Universal SYBR qPCR Mix (Kangyuan Xingye Biotechnology, Beijing, China; Code No. A308-10) on a Bio-Rad CFX96 Real-Time PCR Detection System (USA). Fluorescence signals were monitored in real-time during amplification. Post-amplification, melting curve analysis was performed to verify reaction specificity. ZmActin5 was used as the internal control gene for normalization due to its stable expression across samples. The primers used for ZmActin5 were qActin5-F (GCCGAGCGAGAAATTGTAAG) and qActin5-R (TGGTGATTACTTGGCCATCA) [44]. Relative gene expression was calculated using the 2^(−ΔΔCt)^ method [45]. Three biological replicates with three technical replicates each were included in the experiment.

## Data Availability

The original sequencing data generated in this study have been deposited in the Sequence Read Archive (SRA) database under the accession number PRJNA1378803.

## Supplementary Materials

The following supporting information can be downloaded at https://www.mdpi.com/article/10.3390/plants15010161/s1.

## References

[1] Erenstein O., Jaleta M., Sonder K., Mottaleb K., Prasanna B.M. (2022). Global maize production, consumption and trade: Trends and R&D implications. Food Secur..

[2] Choudhary M., Singh A., Gupta M., Rakshit S. (2020). Enabling technologies for utilization of maize as a bioenergy feedstock. Biofuels Bioprod. Biorefining.

[3] Yang S., Li Y., Wang Y., Zhang X., Liu X. (2025). A natural variant of COOL1 enhances cold tolerance for high-latitude adaptation in maize. Cell.

[4] FAO (2022). Agricultural Production Statistics 2000–2020. FAOSTAT Analytical Brief 41.

[5] Li X., Sun Y., Liu J., Wang Y. (2023). Chilling stress disrupts starch metabolism and redox homeostasis during maize seed germination. Plant Physiol. Biochem..

[6] Zhang J., Huang B. (2012). Chilling-induced oxidative stress and antioxidant responses in maize seedlings. J. Integr. Plant Biol..

[7] Hou P., Li X., Liu Y., Zhang S. (2021). GWAS identifies loci for low-temperature germination ability in maize. Front. Plant Sci..

[8] Fuentes M., Alvarado D., Pinto M. (2020). Cold tolerance evaluation in Chilean rice genotypes at the germination stage. J. Agron. Crop Sci..

[9] Zhao X., Niu Y., Hossain Z., Bai X., Mao T. (2023). Light-quality–regulated plasticity of maize coleoptile and mesocotyl elongation during germination. Front. Plant Sci..

[10] Liu X., Wang Y., Zhang X., Li J., Zhao H., Chen L., Yang M., Zhou Q., Wu S., Sun T. (2020). Identification of cold-tolerant genes in rice via GWAS. Rice Sci..

[11] Shakiba E., Eizenga G.C., McCouch S.R. SNPs Associated with rice seedling cold tolerance identified by GWAS. Proceedings of the Rice Technical Workshop Group.

[12] Zhao X., Wang P., Si T., Hsu C.-C., Wang L., Zayed O., Yu Z., Zhu Y., Dong J., Tao W.A. (2017). MAP kinase cascades regulate the cold response by modulating ICE1 stability. Dev. Cell.

[13] Saijo Y., Hata S., Kyozuka J., Shimamoto K., Izui K. (2000). Overexpression of a Ca^2+^-dependent protein kinase confers cold tolerance in rice. Plant J..

[14] Wang Y., Zhang L., Li J., Zhao H., Chen F. (2020). Wheat TaSOD1.2 maintains redox homeostasis under cold stress. Plant Physiol..

[15] Lee G.H., Kim J., Lee S.C. (2013). Cytosolic ascorbate peroxidase contributes to cold tolerance in rice. Plant Cell Environ..

[16] Tavu L.E.J., Redillas M.C.F.R. (2025). Oxidative Stress in Rice (*Oryza sativa*): Mechanisms, Impact, and Adaptive Strategies. Plants.

[17] Gulzar F., Yang H., Chen J., Hassan B., Huang X., Qiong F. (2024). 6-BA Reduced Yield Loss under Waterlogging Stress by Regulating the Phenylpropanoid Pathway in Wheat. Plants.

[18] Wang C., Hao N., Li Y., Sun N., Wang L., Ye Y. (2025). Cold-tolerance candidate gene identification during maize germination via multi-omics. Agronomy.

[19] Zhang J., Liu J.P., Yang R.J., Yuan Y.Y., Li D.Y., Zhang Z.H. (2019). Melatonin differentially regulates the activities of antioxidant enzymes in roots and coleoptiles of etiolated maize seedlings. J. Plant Growth Regul..

[20] Dou Y., Luo W., Zhang Y., Li W., Zhang C., Lv Y., Liu X., Yu S. (2025). Integrated transcriptome–metabolome analysis reveals the flavonoids metabolism mechanism of maize radicle in response to low temperature. Plants.

[21] Kusvuran S., Kiran S., Ellialtioglu S.S. (2016). Antioxidant enzyme activities and abiotic stress tolerance relationship in vegetable crops. Abiotic and Biotic Stress in Plants—Recent Advances and Future Perspectives.

[22] Vogt T. (2010). Phenylpropanoid biosynthesis. Mol. Plant.

[23] Zhang Y., Li Y., Zhao X., Wang H., Chen J., Liu S., Zhou Q., Wu L., Sun M., Han Z. (2018). Effects of low-temperature stress on seed germination and seedling growth of different maize varieties. J. Northeast Agric. Univ. Engl. Ed..

[24] Li Y., Zhang H., Wang X. (2015). Effects of low temperature on coleoptile growth and cell elongation in wheat. J. Cereal Sci..

[25] Bindschedler L.V., Feussner I., Kwon S.J., Lee J., Choi Y., Apel K., Durner J., Klessig D.F., Park C.M., Kang B.G. (2006). The role of class III peroxidases in plant defence. Trends Plant Sci..

[26] Agati G., Tattini M. (2010). Flavonoids as antioxidants in plants: Location and functional significance. Plant Sci..

[27] Sato Y., Tanaka Y., Nakano Y., Asada K. (2011). Ascorbate peroxidase 2 is a key enzyme for hydrogen peroxide scavenging during photooxidative stress in rice leaves. Plant Cell Physiol..

[28] Qin Y., Li J., Zhang S. (2020). Transcriptome profiling reveals key genes involved in cold stress response in maize seedlings. BMC Genom..

[29] Mao Y., Zhang L., Li J., Wang H., Chen S., Liu Q., Zhao Y., Huang J., Zhou X., Sun W. (2021). Genome-wide association study reveals divergent genetic architectures for cold tolerance at germination and seedling stages in rice. New Phytol..

[30] Li J., Zhang Y., Dong C., Yang S., Wang H., Chen L., Liu Q., Zhao X., Huang M., Zhou T. (2023). Heat shock transcription factor HSF21 enhances cold tolerance in maize by activating the CBF pathway and antioxidant system. Mol. Plant.

[31] He R.Y., Zheng J.J., Chen Y., Pan Z.Y., Yang T., Zhou Y., Li X.-F., Nan X., Li Y.-Z., Cheng M.-J. (2023). QTL-seq and Transcriptomic Integrative Analyses Reveal Two Positively Regulated Genes That Control the Low-Temperature Germination Ability of MTP–Maize Introgression Lines. Theor. Appl. Genet..

[32] Draves M.A., Banerjee A., Eckardt N.A. (2022). Maize seedling growth and hormone response assays using the rolled towel method. Curr. Protoc..

[33] Tandon J.P., Grover S.K., Chaturvedi S.K. (2015). Guidelines for Testing Crop Varieties Under the All-India Coordinated Crop Improvement Projects.

[34] Hakizimana F., Haley S.D., Turnipseed E.B. (2000). Repeatability and genotype × environment interaction of coleoptile length measurements in winter wheat. Crop Sci..

[35] Yan N., Cao J., Wang J., Zou X., Yu X., Zhang X., Si T. (2024). Seed priming with graphene oxide improves salinity tolerance and increases productivity of peanut through modulating multiple physiological processes. J. Nanobiotechnol..

[36] Pruitt K.M., Reiter B., Reiter B., Hjorth S., Tenovuo J. (1985). Biochemistry of peroxidase system: Antimicrobial effects. The Lactoperoxidase System: Chemistry and Biological Significance.

[37] Senthilkumar M., Prasad M.N.V., Sahu P.K., Mandal S.C., Mishra S. (2021). Estimation of malondialdehyde (MDA) by thiobarbituric acid (TBA) assay. Handbook of Stress Tolerance in Plants: Volume 2: Assessment of Plant Stress Tolerance.

[38] Hadi M., Stacy E.A. (2023). An optimized RNA extraction method for diverse leaves of Hawaiian Metrosideros, a hypervariable tree species complex. Appl. Plant Sci..

[39] Anders S., Huber W. (2010). Differential expression analysis for sequence count data. Genome Biol..

[40] Garber M., Grabherr M.G., Guttman M., Trapnell C. (2011). Computational methods for transcriptome annotation and quantification using RNA-seq. Nat. Methods.

[41] Goldstein L.D., Cao Y., Pau G., Lawrence M., Wu T.D., Seshagiri S., Gentleman R. (2016). Prediction and quantification of splice events from RNA-Seq data. PLoS ONE.

[42] Huang J., Wu J., Li C., Xiao C., Wang G. (2009). Specific and sensitive detection of Ralstonia solanacearum in soil with quantitative, real-time PCR assays. J. Appl. Microbiol..

[43] Litholdo C.G., da Fonseca G.C. (2018). Circular RNAs and plant stress responses. Circular RNAs: Biogenesis and Functions.

[44] Jia X., Wang F., Sun B., Zhang X. (2012). Selection of reliable reference genes for quantitative real-time PCR studies in maize grains. Plant Mol. Biol. Report..

[45] Livak K.J., Schmittgen T.D. (2001). Analysis of relative gene expression data using real-time quantitative PCR and the 2^−ΔΔCt^ method. Methods.

