# Supplementary figures and images for: Transcriptomics Reveals Cold Tolerance Maize Lines Involved in the Phenylpropanoid and Flavonoid Pathways
Source: Plants (Basel). 2026 Jan 5;15(1):161. doi: 10.3390/plants15010161 (PMC12787554; doi:10.3390/plants15010161)

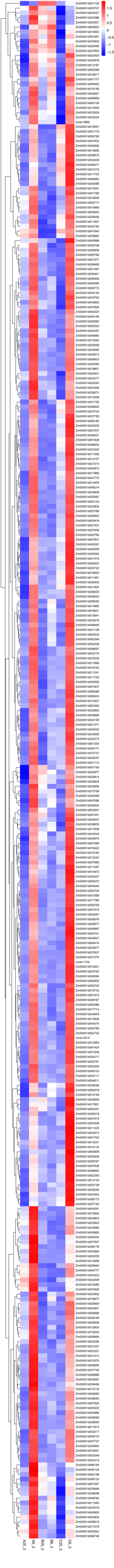

Supplement: Supplementary file 1 [file plants-15-00161-s001.zip › Table S3.pdf]
